# Sciatica due to extrapelvic heterotopic ossification: A case report

**DOI:** 10.1186/1752-1947-2-298

**Published:** 2008-09-10

**Authors:** Elias C Panagiotopoulos, Spyros A Syggelos, Athanasios Plotas, Gregorios Tsigkas, Panagiotis Dimopoulos

**Affiliations:** 1 Department of Orthopaedics, University Hospital of Patras, 26504 Rion Patras, Greece; 2 Department of Radiology, University Hospital of Patras, 26504 Rion Patras, Greece; 3 Department of Cardiology, University Hospital of Patras, 26504 Rion Patras, Greece

## Abstract

**Introduction:**

Sciatica is a common problem, usually caused by disc herniation or spinal stenosis. Low back pain is also present in most cases. When sciatica is the unique clinical finding, especially in young patients, extraspinal pathology should be investigated.

**Case presentation:**

We describe a rare case of sciatica in a 32-year-old man, which was developed as a complication of post-traumatic pelvic heterotopic ossification. During the operation, the sciatic nerve was found to be bluish, distorted and compressed in an hourglass fashion around a heterotopic bone mass. The heterotopic bone tissue, 4 cm in diameter, was removed and the patient had fully recovered 3 months after the operation.

**Conclusion:**

In cases of sciatica without back pain, the possibility of direct pressure of the sciatic nerve from cysts, tumours or bone, as in the present case, should be considered.

## Introduction

Sciatica is defined as pain along the course of the sciatic nerve or its branches and is commonly caused by a herniated disc or spinal stenosis. It is usually combined with low back pain, which affects the lumbosacral region, buttocks and thighs. Symptoms in most cases (up to 85%) are relieved by conservative treatment [1], but surgical treatment may be necessary in persistent cases. Inter-vertebral disc herniation is the most common cause, even in children [2]. Therefore, other causes of sciatic nerve compression, such as infection, tumours, degenerative spine diseases and pelvic pathology may be easily misdiagnosed.

Due to its long path, the sciatic nerve can be compressed in different anatomical areas and by several factors. A rare case of sciatica, in an active young man, due to sciatic nerve pressure by pelvic heterotopic ossification (HO) is reported.

## Case presentation

A 32-year-old active man was referred to our clinic complaining of persistent (4 months duration) numbness of the right lower limb, without any low back pain. The patient had no history of medical problems including symptoms of back pain. The only event, possibly related to the present complaints, was an adductors injury 15 years previously, suffered while playing basketball. The large swelling, which developed at that time, was treated by a few days of bed rest. Since then the patient had been athletically active and he was in training for at least 4 days per week.

The numbness was becoming worse after prolonged sitting and the patient mentioned a feeling of relief while standing or even walking. Training did not affect the intensity of the symptoms.

The Lasegue's sign was positive (in 25° of right hip flexion) and there was a sensory deficit on the dorsal surface of the right foot, to the first interdigital space. Motor weakness or reflex disturbance did not occur and the lumbar spine had a free and painless range of motion. In addition, examination of the hip, pelvis and spine did not indicate any pathology.

**Figure 1:**
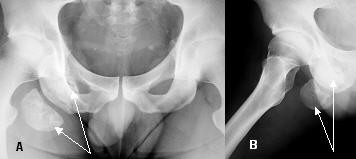
**Pre-operative radiographs of the pelvis**. (A) Anteroposterior; (B) lateral. The arrows point to the heterotopic bony masses.

**Figure 2:**
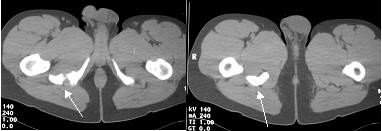
**Pre-operative computed tomography images of the pelvis.** The arrows point to the heterotopic bony masses.

**Figure 3:**
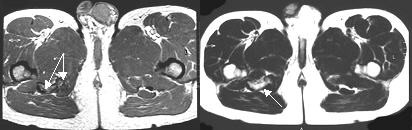
**Pre-operative magnetic resonance imaging scans of the pelvis.** The arrows point to the heterotopic bony masses.

Plain anteroposterior and lateral radiographs of the lumbar and pelvic areas (Figure 1) revealed a normal lumbar spine but also a sizeable global bony mass just below the ischial tuberosity, in contact with a smaller mass. Computed tomography (CT) (Figure 2) and magnetic resonance imaging (Figure 3) scans revealed two heterotopic bone masses within the muscles. Electromyography findings revealed deep peroneal nerve dysfunction accompanied with pathological measurement of f-wave latency. A whole body technetium bone scan was normal.

An operation for nerve exploration and heterotopic bone removal was planned. A posterolateral approach of the right hip was performed. During the procedure, the joint capsule was preserved intact. The sciatic nerve was found to be bluish, distorted and compressed in an hourglass fashion around the larger (4 cm in diameter) heterotopic bony mass. Another smaller bony mass of 4 cm diameter was found behind the nerve. Each mass was enclosed in a fibrous capsule, which, in the case of the larger mass, could not be easily removed because of the presence of strong fibrous bands connected to the sciatic nerve.

Both heterotopic bone masses were dissected and removed. The sciatic nerve was left bluish, lying in a waved manner within the local muscles. Histopathological examination reported the presence of mature bone tissue.

Weight bearing started during the second postoperative day. Symptoms decreased during the third postoperative month and the deep peroneal sensory dysfunction was fully recovered 1 month later. This recovery was documented clinically and by electromyography. The patient received biphosphonates (disodium etidronate, three times daily) for 6 months to avert the recurrence of heterotopic bone formation.

## Discussion

Sciatica affects adults (up to 40%) and may be caused by various intraspinal or extraspinal pathologies. The most common intraspinal cause is inter-vertebral disc prolapse, which can be asymptomatic in 20% of patients. In young patients, the possibility of destructive lesions of osseous or neural tissues of the lumbar spine, such as infection (poliomyelitis, osteomyelitis, spine abscess) or tumours (meningioma, neurofibroma, metastatic lesions), must always be considered in the differential diagnosis. In patients who receive anticoagulants, an intraneural root haemorrhage can also, rarely, occur and a stress fracture could occur due to osteoporosis in those patients receiving corticosteroids. In older patients, degenerative arthritis, which causes narrowing of both the spinal canal and inter-vertebral space, is frequently seen. All of these, less common, intraspinal pathologies can be diagnosed during routine imaging for disc prolapse [3].

Only a few reports on less common intraspinal causes of sciatica have been published. Kao et al. [4] described a lumbar intraspinal ganglion cyst as a cause of spinal stenosis and sciatica. Recently six cases of crystal arthropathy of the lumbar spine, with calcium pyrophosphate dihydrate deposition in the facet joints, generating stenosis and nerve root compression, have been reported [5]. In 2005, Gormus et al. [6] reported an extremely rare case of secondary intraspinal sciatica in a pregnant woman. She had an acute vena cava thrombosis, which generated dilated epidural veins, compressing the neural roots and producing sciatica as a unique symptom.

The extraspinal causes of sciatica are rare. These usually result from pelvic pathology and can easily be missed. Osteophytes at the sacro-iliac joint as a cause of sciatica, because of impingement, have been reported. Pelvic tumours, such as sarcomas, may also cause sciatica and, in most cases, there is a delay in diagnosis with a consequent poor outcome [7]. Piriformis pathology, post-traumatic or infectious, can also generate symptoms of the sciatic nerve because of their anatomical relationship. Furthermore Dosani et al. [8] reported sciatica from nerve pressure by an old avulsed fracture of the ischial tuberosity, which occurred in a 14-year-old girl during a running competition.

We have reported a rare extraspinal case of sciatica in a young man. The sciatic nerve was pushed away and compressed by a large heterotopic bony mass, caused by HO. By definition, HO is the formation of bone within soft tissues. The development of HO is extra-articular and occurs outside the joint capsule. Bone is formed in the connective tissue between the muscle planes and not within the muscle itself [9]. New bone can be contiguous to the skeleton but generally does not involve the periosteum [9].

The signs and symptoms of HO are non-specific and diagnosis in the initial stages is difficult. In this early inflammatory phase, the condition may mimic cellulitis, thrombophlebitis, osteomyelitis or tumour [9]. Later, reduced range of motion and ankylosis of the joint may occur.

The typical radiological appearance of HO, similar to that shown in our patient's pre-operative radiographs, is a circumferential ossification with a lucent centre. CT can provide a more accurate three-dimensional localisation of the ossification and may reveal heterotopic bone that has not been detected by plain film radiography.

The prevention of HO has been widely investigated, especially in patients who have had total hip replacement. Prophylaxis against HO primarily includes local radiation, non-steroidal anti-inflammatory drugs and biphosphonates [10]. Finally, the unique effective treatment for established heterotopic bone is surgical resection, although its recurrence is a problem.

## Conclusion

In cases of sciatica, especially without back pain, the possibility of direct extraspinal pressure on the sciatic nerve caused by cysts, tumours or bone, as in the present case, should be considered.

## Abbreviations

CT: Computed Tomography; HO: Heterotopic Ossification.

## Competing interests

The authors declare that they have no competing interests.

## Consent

Written informed consent was obtained from the patient for publication of this case report and any accompanying images. A copy of the written consent is available for review by the Editor-in-Chief of this journal.

## Authors' contributions

EP and SAS treated the patient. AP and PD performed the imaging tests. GT was responsible for the pre-operative preparation of the patient. All authors read and approved the final manuscript.
